# Time- and dose-dependent effects of total-body ionizing radiation on muscle stem cells

**DOI:** 10.14814/phy2.12377

**Published:** 2015-04-13

**Authors:** Shinya Masuda, Tsubasa Hisamatsu, Daiki Seko, Yoshishige Urata, Shinji Goto, Tao-Sheng Li, Yusuke Ono

**Affiliations:** Department of Stem Cell Biology, Atomic Bomb Disease Institute, Nagasaki University Graduate School of Biomedical SciencesNagasaki, Japan

**Keywords:** Hormesis, low-dose irradiation, muscle stem cells, satellite cells, skeletal muscle, tissue stem cells

## Abstract

Exposure to high levels of genotoxic stress, such as high-dose ionizing radiation, increases both cancer and noncancer risks. However, it remains debatable whether low-dose ionizing radiation reduces cellular function, or rather induces hormetic health benefits. Here, we investigated the effects of total-body *γ*-ray radiation on muscle stem cells, called satellite cells. Adult C57BL/6 mice were exposed to *γ*-radiation at low- to high-dose rates (low, 2 or 10 mGy/day; moderate, 50 mGy/day; high, 250 mGy/day) for 30 days. No hormetic responses in proliferation, differentiation, or self-renewal of satellite cells were observed in low-dose radiation-exposed mice at the acute phase. However, at the chronic phase, population expansion of satellite cell-derived progeny was slightly decreased in mice exposed to low-dose radiation. Taken together, low-dose ionizing irradiation may suppress satellite cell function, rather than induce hormetic health benefits, in skeletal muscle in adult mice.

## Introduction

Accumulating evidence suggests that exposure to high levels of ionizing radiation results in DNA damage and consequent chromosomal rearrangements (Huang et al. 2003), leading to increases in both cancer and noncancer risks. Tissue stem cells comprise a small cellular population with important roles in repair, regeneration, and homeostasis in adult tissues. Thus, alterations in the quality and/or quantity of tissue stem cells may have potential as a predictive risk indicator for future health hazards (Prise and Saran 2011).

Generally, low-dose ionizing radiation is defined as a dose between background radiation (0.01 mSv/day) and high-dose radiation (>150 mSv/day) (Bonner 2003). To date, the biological and pathological effects of ionizing radiation on humans have been evaluated by epidemiological studies. The Life Span Study on a cohort of atomic bomb survivors revealed a significant increase in cancer at doses above 100 mGy, but no evidence at lower doses (Huang et al. 2003). However, a recent study on CT scans in children suggested that cumulative doses of approximately 50 and 60 mGy might increase the risks of leukemia and brain cancer, respectively (Preston et al. 2007). Following the 2011 incident at the Fukushima Daiichi nuclear power plant in Japan, it has become urgently necessary to understand how exposure to low-dose radiation influences human health (Fushiki 2013; Suzuki et al. 2015; Walsh et al. 2014; Yabe et al. 2014).

Although humans are constantly exposed to low levels of natural ionizing radiation in the environment, the health effects of low-dose ionizing radiation are still under debate (Huang et al. 2003). According to the “radiation hormesis hypothesis”, exposure to low-dose irradiation may induce biological health benefits through cellular adaptive responses (Sanderson and Morley 1986; Moquet et al. 1989; Shadley and Dai 1993; Park et al. 1999; Raaphorst and Boyden 1999). For example, low-dose irradiation stimulated proliferation and improved cell-survival adaptations to subsequent radiation exposure in splenocytes in mice (Yoshida et al. 1993; Hyun et al. 1997). However, there is little evidence on the effects of low-dose ionizing radiation on tissue stem cells (Manda et al. 2014).

The resident skeletal muscle stem cells, called satellite cells, are located between the basal lamina and the plasmalemma of myofibers (Mauro 1961). Satellite cells are responsible for providing myonuclei for postnatal muscle growth, hypertrophy, repair, and regeneration in adults (Otto et al. 2009; Relaix and Zammit 2012; Ono 2014; Wang et al. 2014). These cells generate myoblasts, which are muscle progenitors that undergo extensive proliferation before fusing with one another to form myotubes that eventually become mature myofibers or fusing with existing myofibers. In healthy muscle, satellite cells are mitotically quiescent and express the paired-box transcription factor Pax7 (Seale et al. 2000; Zammit et al. 2006; Lepper et al. 2011). Meanwhile, satellite cells are activated in response to stimulation such as physical activity and traumatic muscle injury. Activated satellite cells upregulate MyoD, belonging to a family of myogenic regulatory factors (MRFs), become myoblasts, and undergo proliferation. Following extensive proliferation, the majority of satellite cell-derived myoblasts downregulate Pax7, maintain MyoD, express myogenin (another MRF family member), and undergo myogenic differentiation to generate myosin heavy-chain (MyHC)-positive new myonuclei. A minority population of cells downregulate MyoD, maintain Pax7 expression, and return to a quiescent state for self-renewal to maintain the stem cell pool (Halevy et al. 2004; Olguin and Olwin 2004; Zammit et al. 2004; Collins et al. 2005; Montarras et al. 2005; Sacco et al. 2008). The total number of satellite cells in adult muscle remains relatively constant after repeated muscle injury and regeneration throughout life. Thus, satellite cell self-renewal is considered to be carefully regulated (Kuang et al. 2007; Ono et al. 2010, 2012; Sambasivan et al. 2011; Chakkalakal et al. 2012, 2014).

In this study, we exposed adult mice to different doses of *γ*-ray radiation (2–250 mGy/day) for 30 days, and investigated the sensitivity and dose-dependency of the radiation effects on muscle stem cells at the acute and chronic phases following radiation exposure.

## Materials and Methods

### Antibodies and reagents

Mouse anti-Pax7, mouse anti-Myogenin, and rabbit anti-MyoD antibodies were purchased from Santa Cruz Biotechnology (Santa Cruz, CA). The mouse anti-MyHC (MF20) antibody was purchased from R&D Systems (Minneapolis, MN). The rat anti-Ki67 antibody was purchased from DAKO (Glostrup, Denmark). Mounting medium containing DAPI for nuclear staining was purchased from Vector Laboratories (Burlingame, CA).

### Animals and total-body *γ*-ray radiation

Twelve-week-old C57BL/6 male mice (SLC, Tokyo, Japan) were used. All animal experiments were approved by the Experimental Animal Care and Use Committee of Nagasaki University, and were performed in accordance with institutional and national guidelines. Total-body *γ*-radiation was performed by daily exposure of the mice to 0, 2, 10, 50, and 250 mGy for 30 days, with cumulative doses of 0, 60, 300, 1500, and 7500 mGy, respectively. The irradiated mice were killed immediately (acute exposure phase) or at 3 months (chronic exposure phase) after the last exposure. The body weight was not significantly different among conditions at both acute and chronic phases after the radiation as previously described (Guo et al. 2015).

### Satellite cell isolation and culture

Individual myofibers associated with satellite cells were isolated from EDL muscles and digested with type I collagenase, as previously described (Ono et al. 2015). Satellite cells were obtained from isolated myofibers by trypsinization in a 0.125% trypsin-EDTA solution for 10 min at 37°C under 5% CO_2_. The satellite cells were cultured in GM (GlutaMax DMEM supplemented with 30% FBS, 1% chicken embryo extract, 10 ng/mL bFGF, and 1% penicillin-streptomycin) at 37°C under 5% CO_2_. Myogenic differentiation was induced by culture in DM (GlutaMax DMEM supplemented with 5% horse serum and 1% penicillin-streptomycin) at 37°C under 5% CO_2_.

### Immunostaining

Immunocytochemistry of satellite cells and isolated single myofibers was performed as described previously (Ono et al. 2010). Samples were blocked and permeabilized by incubation with phosphate-buffered saline containing 0.3% Triton X100 and 5% goat or swine serum for 20 min at room temperature, and then incubated with primary antibodies at 4°C overnight. All immunostained samples were visualized using appropriate species-specific Alexa Fluor 488- and/or 568-conjugated secondary antibodies (Life Technologies, Tokyo, Japan). Digital images were acquired with a DP80 camera using cellSens software (Olympus, Tokyo, Japan). Images were optimized globally and assembled into figures using Adobe Photoshop.

### Statistical analysis

The significance of differences among data was determined using Student's *t*-test. Values of *P* < 0.05 were considered to indicate statistical significance.

## Results

**Figure 1:**
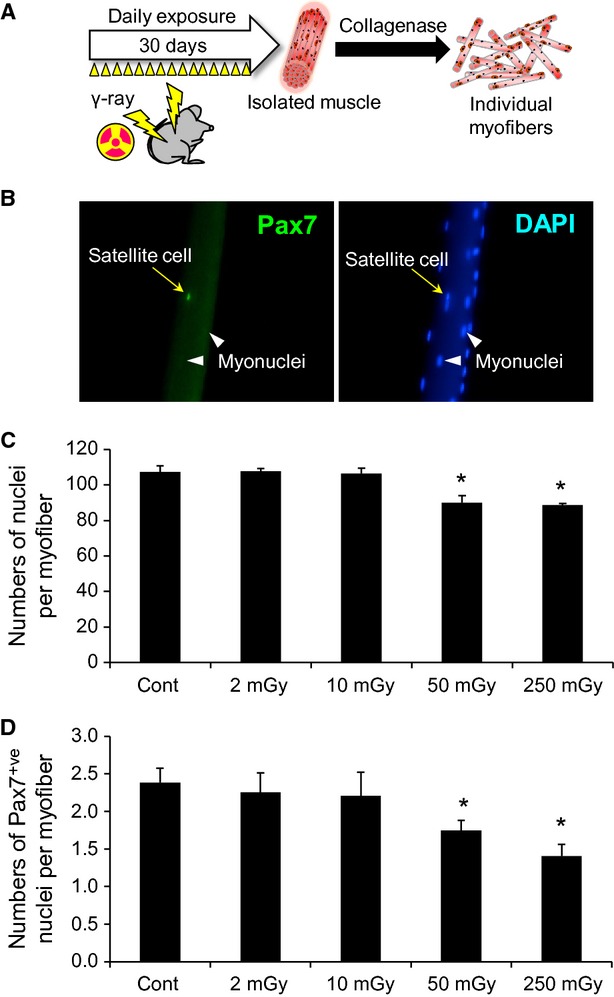
Numbers of myonuclei and satellite cells are both decreased with exposure to moderate- and high-dose *γ*-ray irradiation. (A) Schedule of the total-body *γ*-ray irradiation. Adult mice were exposed to daily *γ*-ray irradiation at low- to high-dose rates (low, 2 or 10 mGy/day; moderate, 50 mGy/day; high, 250 mGy/day) for 30 days. The mice were killed immediately after the last irradiation and individual myofibers associated with satellite cells were freshly isolated from EDL muscles by collagenase digestion. (B–D) Satellite cells and myonuclei were visualized by immunostaining with an anti-Pax7 antibody and DAPI staining in isolated individual myofibers associated with satellite cells (quantified in C and D, respectively). Data represent means ± SEM (*n* = 6 mice). *P < 0.05, significant difference from control mice.

It remains largely unknown whether low-dose ionizing radiation can induce hormetic responses in tissue stem cells (Manda et al. 2014), especially in noncarcinogenic tissues such as skeletal muscle. In this study, total-body *γ*-radiation was performed by daily exposure of C57BL/6 adult mice to 0, 2, 10, 50, and 250 mGy doses for 30 days, with cumulative doses of 0, 60, 300, 1500, and 7500 mGy, respectively (Bonner 2003). The doses of ionizing radiation were classified according to a previous report (Kadhim et al. 2013): low-dose radiation (LDR; 2 or 10 mGy/day); moderate-dose radiation (MDR; 50 mGy/day); high-dose radiation (HDR; 250 mGy/day). The irradiated mice were killed immediately (acute exposure phase) or at 3 months (chronic exposure phase) after the last exposure (Fig.1A).

### Numbers of both myonuclei and satellite cells in muscle are decreased by exposure to moderate and high doses of total-body *γ*-irradiation

First, we investigated whether total-body *γ*-ray irradiation affects myofibers and satellite cells. Myofibers associated with satellite cells were freshly isolated from extensor digitorum longus (EDL) muscles. Satellite cells were visualized by immunostaining with an anti-Pax7 antibody and the total nuclei per myofiber were evaluated by the number of DAPI^+ve^ nuclei. The numbers of both myonuclei and satellite cells were reduced with MDR (50 mGy) and HDR (250 mGy), but not LDR (2 and 10 mGy) (Fig.1B and C).

### Exposure to moderate and high doses of irradiation impairs population expansion of satellite cells

**Figure 2:**
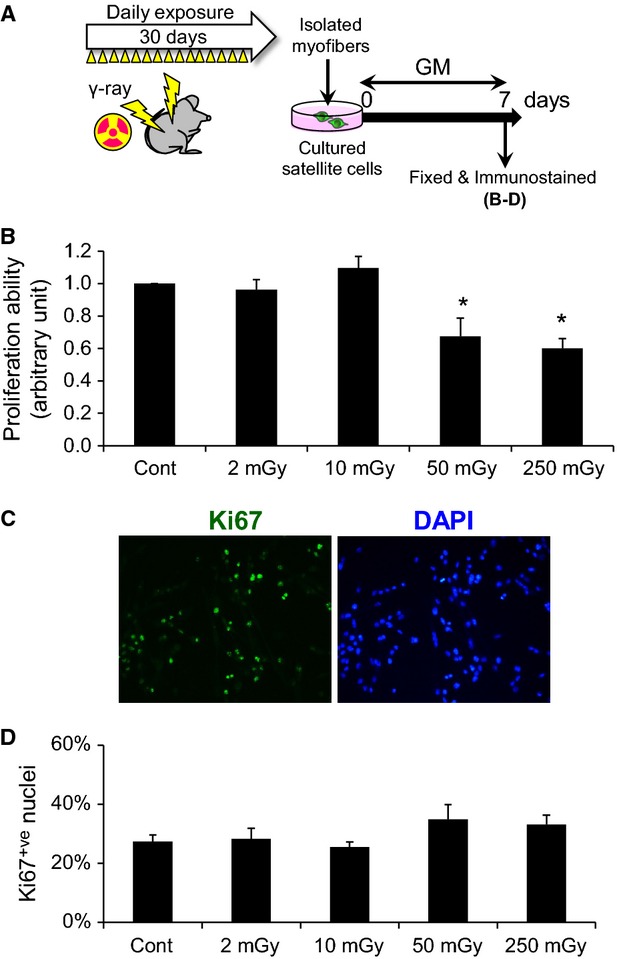
Exposure to moderate- and high-dose *γ*-ray irradiation impairs population expansion of satellite cells. (A) Adult mice were exposed to daily *γ*-ray irradiation for 30 days as shown in Figure1. The mice were killed immediately after the last irradiation, and individual myofibers associated with satellite cells were freshly isolated from EDL muscles by collagenase digestion. The isolated myofibers were cultured in GM for 7 days. (B) Isolated satellite cells were cultured in GM for 7 days. Proliferation ability of satellite cells was evaluated by counting the total number of DAPI-stained nuclei. (C) Immunostaining for a proliferation marker, Ki67, in satellite cells (quantified in D). Data represent means ± SEM (*n* = 6 mice). *P < 0.05, significant difference from control mice.

Next, we examined the effects of total-body *γ*-ray irradiation on the proliferation ability of satellite cells. Satellite cells are mitotically quiescent and express Pax7. Upon activation by muscle injury, satellite cells upregulate MyoD together with Pax7 and undergo proliferation. Satellite cells associated with myofibers were freshly isolated from EDL muscles and cultured in growth medium (GM) for 7 days following exposure to *γ*-ray irradiation (Fig.2A). The proliferation ability of satellite cells was evaluated by counting the total number of DAPI^+ve^ satellite cell-derived cells. Although exposure to MDR and HDR significantly suppressed the population expansion of satellite cells (Fig.2B), immunostaining for Ki67, a proliferation marker, showed that the percentage of Ki67^+ve^ nuclei was unchanged (Fig.2C, quantified in Fig.2D). These data suggest that *γ*-ray irradiation at MDR and HDR lowers the ability of population expansion with a delay in cell-cycle progression in satellite cells.

### Myogenic differentiation is not affected by *γ*-ray irradiation

**Figure 3:**
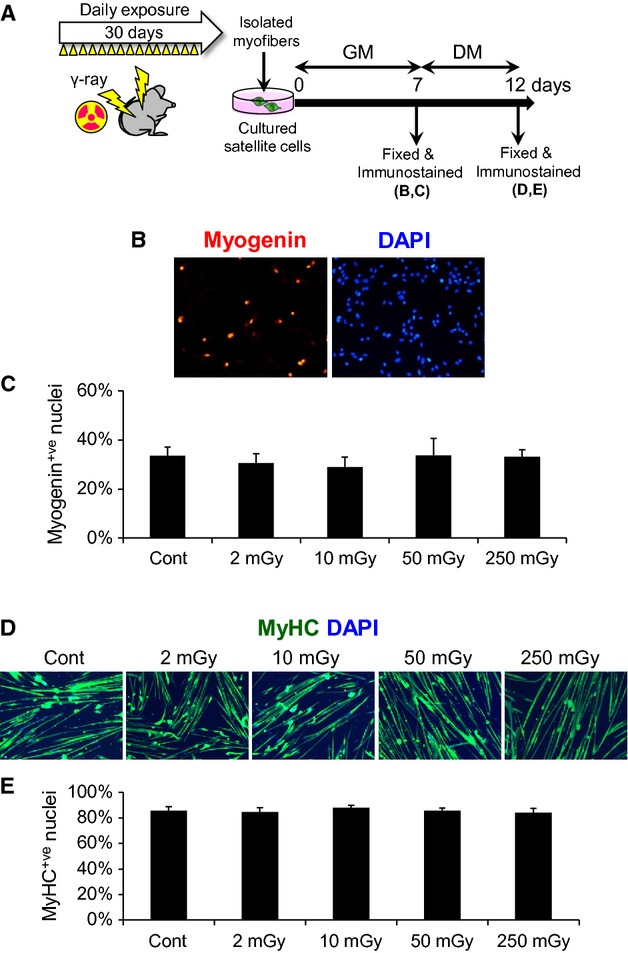
Differentiation is unaffected by *γ*-ray irradiation in satellite cells. (A–E) Satellite cells associated with single myofibers were isolated from EDL muscles of mice with daily exposure to *γ*-ray irradiation for 30 days as shown in Figure1, and maintained in GM for 7 days (B, C) before induction of myogenic differentiation by culture in DM (D, E). (B) Immunostaining for myogenin, a marker of myogenic differentiation (quantified in C). (D) Immunostaining for MyHC (quantified in E). Data represent means ± SEM (*n* = 6 mice).

Given that the proliferation ability was impaired by exposure to MDR and HDR, we further determined whether myogenic differentiation was also affected by the irradiation. Myogenic differentiation of satellite cells was induced by culture in differentiation medium (DM) for 5 days following extensive proliferation as described for Figure2A (Fig.3A). Immunostaining for myogenin, which is expressed in cells committed to differentiation, revealed that the differentiation ability was unchanged by the exposure to *γ*-ray irradiation (Fig.3B, quantified in Fig.3C). To further determine the late stage of myogenic progression, freshly isolated satellite cells were induced to differentiate by culture in DM for 5 days and stained with an anti-MyHC antibody. We confirmed that irradiated satellite cells were able to form multinucleated myotubes and express MyHC in the late stage of differentiation (Fig.3D, quantified in Fig.3E). Thus, these data indicate that myogenic differentiation is unaltered by total-body *γ*-ray irradiation.

### Satellite cell self-renewal is upregulated by *γ*-ray irradiation

**Figure 4:**
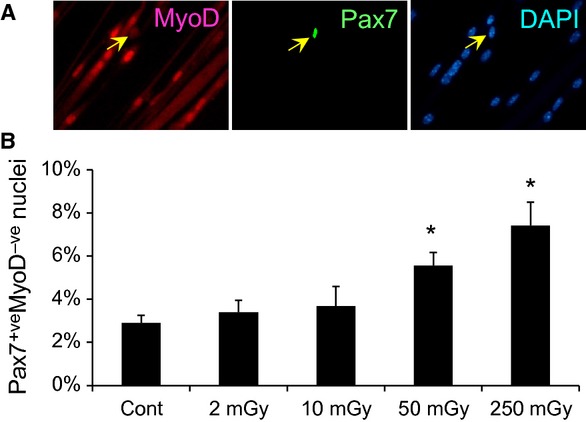
Self-renewal ability is maintained after *γ*-ray irradiation. To investigate whether self-renewal is affected by *γ*-ray irradiation, satellite cells were isolated from EDL muscles of mice exposed to daily *γ*-ray irradiation for 30 days and maintained in GM for 7 days before induction of myogenic differentiation by culture in DM for 5 days as shown Figure3. (A) Immunocytochemistry for Pax7 and MyoD to visualize Pax7^+ve^MyoD^−ve^ self-renewing cells (arrows; quantified in B). Data represent means ± SEM (*n* = 6 mice). *P < 0.05, significant difference from control mice.

To evaluate whether total-body *γ*-irradiation influences satellite cell self-renewal, plated satellite cells were induced to differentiate by switching to DM following culture in GM as described in Figure3A. Satellite cell self-renewal was determined by immunofluorescence staining for Pax7 and MyoD proteins, as characterized elsewhere (Halevy et al. 2004; Zammit et al. 2004; Ono et al. 2011). Immunocytochemistry revealed that the percentage of Pax7^+ve^MyoD^−ve^ self-renewing cells was increased by exposure to MDR and HDR, but not LDR, in a dose-dependent manner (Fig.4A**,** quantified in Fig.4B), which mirrored the proliferation ability shown in Figure2B. Thus, *γ*-ray irradiation-induced genotoxic stress may promote self-renewal of satellite cells to maintain a stem cell pool in skeletal muscle.

### Satellite cell proliferation is impaired even with low-dose irradiation at the chronic phase

**Figure 5:**
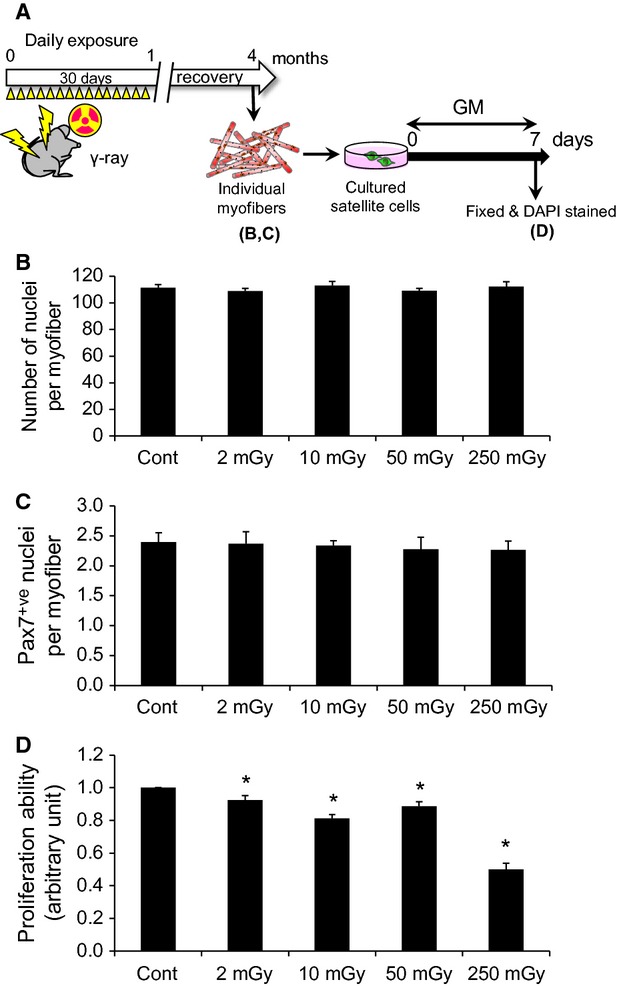
Proliferation ability is impaired by even low-dose *γ*-irradiation in the chronic phase. (A) Schedule of the total-body *γ*-ray irradiation. To examine the effects of *γ*-irradiation on satellite cells in the chronic exposure phase, mice were killed at 3 months after the last *γ*-irradiation exposure. Individual myofibers associated with satellite cells were freshly isolated from EDL muscles, and satellite cells were cultured in GM for 7 days as shown in Figure2. (B, C) Numbers of DAPI^+ve^ myonuclei and Pax7^+ve^ satellite cells per myofiber (quantified in B and C, respectively). (D) The proliferation ability was evaluated by counting the total number of DAPI^+ve^ nuclei for satellite cells. Data represent means ± SEM (*n* = 6 mice). *P < 0.05, significant difference from control mice.

Finally, to examine the effect of exposure to total-body *γ*-ray irradiation on satellite cells and myofibers at the chronic phase, irradiated mice were killed at 3 months after the last irradiation and single myofibers associated with satellite cells were freshly isolated from EDL muscles as shown in Figure1 (Fig.5A). Satellite cells were visualized by immunostaining with an anti-Pax7 antibody and the total nuclei per myofiber were evaluated by the number of DAPI^+ve^ nuclei. The immunostaining analysis revealed that the reduced numbers of Pax7^+ve^ cells and myonuclei observed after MDR and HDR exposure at the acute phase (Fig.1C and D) returned to the basal levels at the chronic phase (Fig.5B and C). Surprisingly, however, the proliferation ability was slightly decreased, even for LDR, at the chronic exposure phase (Fig.5D). Taken together, our results demonstrate that, in adult skeletal muscle, exposure to even a low-dose of *γ*-ray irradiation influences satellite cell function at the chronic phase, rather than inducing hormetic health benefits.

## Discussion

Tissue stem cells in adults retain the ability to proliferate, self-renew, and differentiate throughout life to maintain tissue homeostasis and repair injuries. Understanding how the balance between self-renewal and differentiation is regulated in satellite cells is central to elucidating how skeletal muscle maintains a stem cell pool and homeostasis throughout life (Relaix and Zammit 2012; Ono 2014; Wang et al. 2014). In this study, we investigated the time- and dose-dependent effects of ionizing irradiation on adult muscle satellite cells, which are normally maintained in a quiescent state, at the acute and chronic exposure phases. We found that the numbers of both myonuclei and satellite cells per myofiber were decreased after MDR or HDR, but not LDR, at the acute exposure phase. Unexpectedly, the MDR and HDR exposures also resulted in increased self-renewal of satellite cells in a dose-dependent manner, whereas myogenic differentiation was unaltered. Thus, our data indicate that moderate and high levels of ionizing radiation upregulate the self-renewal ability of satellite cells to maintain the stem cell pool in muscle even with reduced proliferative capacity.

We found that the proliferation, differentiation, and self-renewal of satellite cells were unaffected by LDR at the acute phase. Surprisingly, however, the proliferative ability of satellite cells was slightly decreased in LDR-exposed mice at the chronic phase. This unexpected result indicates that LDR exposure can negatively influence the function of satellite cells, rather than inducing hormetic health benefits, in skeletal muscle of adult mice. Consistent with our findings, exposure of mice to total-body *γ*-irradiation for a long period (approximately 400 days) at a very low-dose rate (1.1 mGy/day) resulted in a shortened lifespan in female mice, but not in male mice, although there was no incidence of tumors (Tanaka et al. 2003).

Our results showed that the numbers of both myonuclei and satellite cells per myofiber were decreased with MDR or HDR at the acute exposure phase. Although these reductions were completely restored to the control levels at the chronic exposure phase, the ability for population expansion of satellite cells was still decreased. These results suggest that better understanding of the effects of total-body *γ*-ray irradiation is necessary to evaluate not only the quantity, but also the quality, of tissue stem cells in the acute and chronic exposure phases.

One of the well-investigated tissue stem cell types on radiosensitivity is hematopoietic stem cells (Heylmann et al. 2014). Recently, we examined the sensitivity and dose-dependency of radiation-induced injury in hematopoietic stem/progenitor cells in mice with daily exposure under the same conditions used in this study (Guo et al. 2015). The percentages of CD34^+ve^ hematopoietic stem/progenitor cells isolated from bone marrow were reduced in mice exposed to even LDR at the acute phase. This reduction was fully recovered for LDR and MDR, but not for HDR, at the chronic phase (Guo et al. 2015). Taken together, these data indicate that the sensitivity to total-body *γ*-ray irradiation depends on the specific stem cell type involved.

In this study, we examined the effects of exposure to total-body *γ*-ray radiation on muscle tissue stem cells. No hormetic responses in proliferation, differentiation, or self-renewal of satellite cells at the acute phase were observed for LDR, which may influence the proliferative ability of satellite cells at the chronic phase. In conclusion, LDR may attenuate satellite cell function, rather than induce hormetic health benefits, in skeletal muscle. In this study, we were not able to undertake the effect of exposure to ionizing radiation on muscle regeneration in vivo. Future studies will be valuable to understand the effects of low levels of ionizing irradiation on tissue stem cells, including satellite cells, in vivo as well as in vitro.
